# A multiple breast cancer stem cell model to predict recurrence of T_1–3_, N_0_ breast cancer

**DOI:** 10.1186/s12885-019-5941-5

**Published:** 2019-07-24

**Authors:** Yan Qiu, Liya Wang, Xiaorong Zhong, Li Li, Fei Chen, Lin Xiao, Fangyu Liu, Bo Fu, Hong Zheng, Feng Ye, Hong Bu

**Affiliations:** 1 Laboratory of Pathology, West China Hospital, Sichuan University, Chengdu, China; 2 Key Laboratory of Transplant Engineering and Immunology, Ministry of Health, West China Hospital, Sichuan University, Chengdu, China; 3 Clinical Research Center for Breast, West China Hospital, Sichuan University, Chengdu, China; 4 Department of Pathology, West China Hospital, Sichuan University, Chengdu, China; 5 Big Data Research Center, School of Computer Science and Engineering, University of Electronic Science and Technology of China, Chengdu, China; 6 Laboratory of Molecular Diagnosis of Cancer & Cancer Center, West China Hospital, Sichuan University, Chengdu, China; 7 West China School of Medicine, Sichuan University, Chengdu, China

**Keywords:** Early stage breast cancer, Brest cancer stem cell, Relapse risk score, Prognosis

## Abstract

**Background:**

Local or distant relapse is the key event for the overall survival of early-stage breast cancer after initial surgery. A small subset of breast cancer cells, which share similar properties with normal stem cells, has been proven to resist to clinical therapy contributing to recurrence.

**Methods:**

In this study, we aimed to develop a prognostic model to predict recurrence based on the prevalence of breast cancer stem cells (BCSCs) in breast cancer. Immunohistochemistry and dual-immunohistochemistry were performed to quantify the stem cells of the breast cancer patients. The performance of Cox proportional hazard regression model was assessed using the holdout methods, where the dataset was randomly split into two exclusive sets (70% training and 30% testing sets). Additionally, we performed bootstrapping to overcome a possible biased error estimate and obtain confidence intervals (CI).

**Results:**

Four groups of BCSCs (ALDH1A3, CD44^+^/CD24^−^, integrin alpha 6 (ITGA6), and protein C receptor (PROCR)) were identified as associated with relapse-free survival (RFS). The correlated biomarkers were integrated as a prognostic panel to calculate a relapse risk score (RRS) and to classify the patients into different risk groups (high-risk or low-risk). According to RRS, 67.81 and 32.19% of patients were categorized into low-risk and high-risk groups respectively. The relapse rate at 5 years in the low-risk group (2.67, 95% CI: 0.72–4.63%) by Kaplan-Meier method was significantly lower than that of the high-risk group (19.30, 95% CI: 12.34–26.27%) (*p* <  0.001). In the multiple Cox model, the RRS was proven to be a powerful classifier independent of age at diagnosis or tumour size (*p* <  0.001). In addition, we found that high RRS score ER-positive patients do not benefit from hormonal therapy treatment (RFS, *p* = 0.860).

**Conclusion:**

The RRS model can be applied to predict the relapse risk in early stage breast cancer. As such, high RRS score ER-positive patients do not benefit from hormonal therapy treatment.

**Electronic supplementary material:**

The online version of this article (10.1186/s12885-019-5941-5) contains supplementary material, which is available to authorized users.

## Background

More than 50% of patients with breast cancer are classified into the early-stage (T_1–3_N_0_M_0_) group [1]. Despite systemic adjuvant therapy dramatically increasing the clinical outcome of patients with early breast cancer, relapse still occurs in more than 20% of patients after surgery within 10 years [2]. Relapse, including recurrence both at local or distant sites, is the main cause for patient deaths, and thus remains an unmet challenge for a curative treatment of breast cancer. It is pivotal to identify patients at risk of relapse at early stages in hopes of improving clinical outcomes, especially within the subgroup of node-negative females, defined as a relatively indolent disease based on pathologic features. Recently, several multigene assays have been developed for early-stage breast cancer patients [3]. Multigene assays are able to provide more prognostic information than traditional parameters in several tumour types [4–11], and several of them have been adopted by the oncology guidelines for treatment. One example is 21-gene expression profiling, which has been widely accepted in clinical practice [12].

As reported, breast cancer is a tumour with high heterogeneity. Although recent advancements have further divided this heterogeneous disease into distinct subgroups by gene expression profiling (GEP) assays, among other methods, several intriguing findings revealed that a small subset of cells isolated from different subgroups of breast cancers exhibit remarkable similar biological behaviours. These subset of cells were defined as cancer stem cells (CSCs) and reported to be responsible for the heterogeneity. Accumulating evidence has proved that CSCs retain the critical characteristics of normal stem cells, such as ability self-renewal and the capacity of proliferation, which contribute significantly to therapeutic resistance and breast cancer relapse [13–17]. In addition, several articles indicated that some CSCs might be derived from normal stem cells, which suggested that normal mammary stem cells might share similar identifying markers [18–20]. Mammary stem cell markers or combined markers have been certified in different stages of stem cells in breast cancer, including ALDH, CD44, CD24, ITGA6/EpCAM, and PROCR. [21–26]. Some of these markers and combined markers (i.e., CD44^+^/CD24^low^ ALDH^+^ and ITGA6^+^) are considered to correlate with poor prognosis in breast cancer [21, 27, 28], because they also identified a BCSC subpopulation [14, 21, 26, 29]. In addition, it has been suggested that ITGA6^+^/EpCAM^+^ mammary luminal progenitor cells were possible transformation targets in basal-like breast cancers, which have close associations with poor prognosis. In addition, it was reported that ITGA6 may define the mesenchymal population and is necessary for CSC function [30–32]. PROCR was reported to be highly expressed in myoepithelial cells of the mammary gland. In a recent study, Wang D et al. identified PROCR as a marker of multipotent mammary stem cells. They found that PROCR-positive mammary cells exhibited epithelial-to-mesenchymal transition (EMT) characteristics, and had high tumorigenesis ability in vivo, which suggested that PROCR-positive mammary cells might be one of the progenitor populations for breast CSCs (BCSCs) [24]. Furthermore, PROCR also promotes tumour metastasis in cancer cell lines [33, 34].

To explore the prognostic role of mammary stem cell (MSCs) and BCSC markers, we have studied the ALDH family (including ALDH1A1, ALDH1A3, ALDH3A1, ALDH4A1, ALDH6A1, and ALDH7A1), PROCR, and ITGA6/EpCAM. In a medium cohort of patients in previous studies, these findings revealed that ALDH1A3, PROCR, ITGA6^+^, ITGA6^+^/EpCAM^−^ and ITGA6^−^/EpCAM^+^ were correlated with reduced RFS or overall survival of these breast cancer patients [35–37]. In this study, we defined these markers and CD44^+^/CD24^low^ as BCSC-associated markers and employed these biomarkers to label stem cells among patients with early stage breast cancer. ALDH1A3, CD44^+^/CD24^−^, ITGA6, and PROCR were shown to be closely associated with RFS. Then, they were integrated into the prognostic panel to calculate an RRS. Patients were then divided into two distinct risk groups, which effectively shows promise in predicting prognosis and treatment. In addition, several EMT transition associated markers, proliferation factors and other clinicopathological parameters were also included in our study to improve the efficiency of our model.

## Materials and methods

### Breast cancer patient dataset

Clinical information from 1036 patients with breast invasive ductal carcinoma (BIDC) diagnosed from 2006 to 2011 was collected from West China Hospital. After selection, 407 patients were enrolled into our study. All the patients were adult females and were treated with mastectomy or lumpectomy to negative margins and with axillary lymph node dissection. Axillary nodes of patients were observed to be without metastasis under microscope. Patients with local invasion and distant metastasis identified initially were ineligible. Patients with neoadjuvant chemotherapy were removed from our study group to avoid its impact on the characteristics of tumour cells in paraffin embedded tissues. Patients enrolled in the study were considered to be early-stage BIDC and defined as entire datasets. The end-point of follow-up was occurrence of local recurrence or distant metastasis. Detailed information of this dataset is listed in Additional file 4: Table S1.

### Breast cancer stem cell biomarkers

BCSC-associated biomarkers were selected from literature as well as our previously confirmed biomarkers including CD44^+^/CD24^−^, ALDH1A3, EpCAM/ITGA6, and PROCR, which showed prognostic value in BIDC [21, 27, 28, 35–37].

### Immunohistochemistry (IHC)

Single staining of CD44, CD24, EpCAM, ITGA6, ALDH1A3, PROCR, Twist and Slug were performed with the EnVision Staining System, while dual staining of CD44/CD24 and EpCAM/ITGA6 were performed with the EnVision G | 2 Doublestain System. The haematoxylin and eosin (H&E) staining, as well as the results of IHC staining were observed under bright field microscopy. Pathological assessment of the tumours were conducted by pathologists at West China Hospital anonymously, including subtypes, histological grades, oestrogen receptor (ER), progesterone receptor (PR), and human epidermal growth factor receptor 2 (HER2) etc. HER2 staining was analysed according to the guidelines of the American Society of Clinical Oncology. ER and PR were analyzed by Allred system [38, 39]. The scoring of BCSC-associated markers, such as ALHD1A3, PROCR, ITGA6, CD44/CD24 and EpCAM/ITGA6 were performed as follows: 0, 0% positive tumour cells; 1, 1 to 10% positive cells; 2, 11 to 50% positive cells; 3, 51 to 75% positive cells; and 4, 76 to 100% positive cells [27]. Scores of Twist and Slug were interpreted as follows: the percentage (P) of positive cells (score 0 for 0%, 1 for ≤1%, 2 for 1–10%, 3 for 10–33%, 4 for 33–66%, and 5 for 66–100% positive cells) and the intensity (I) of staining (score 0 for negative, 1 for weak, 2 for moderate, and 3 for strong staining) were included. A Quick score was generated. (Q = P*I; score range, 0–12) [40].

Detailed information and specificity of these antibodies were shown in Additional file 5: Table S2, Additional file 1: Figure S1, respectively.

### Statistical analysis and model construction

The associations between relapse-free survival (RFS) and the expression panel were analysed by the Cox proportional hazard regression model [41]. To investigate the effectiveness of the BCSC-associated biomarker panel for clinical outcome prediction, we assigned each patient a risk score according to a linear combination of the expression level of BCSC-associated markers. The RRS for sample i using the information from the significant biomarkers was calculated as follows: $\mathrm{RRS}={\sum}_{\mathrm{j}=1}^4\mathrm{Wj}\ast \mathrm{Sj}.$ In the above formula, Sj is the IHC score for biomarker j, and Wj is the weight of the IHC score of biomarker j. Weights were obtained by the coefficients derived from the univariate Cox proportional hazard regression [42]. The RRS was calculated out by the receiver operating characteristic curve (ROC, non-parametric test), which identifies the cut-off value based on the maximum sums of specificity and sensitivity in the ROC curve. Meanwhile, to investigate the association between the relapse and other clinicopathological variables, univariate Cox proportional hazard regression analysis was adopted using clinicopathological factors (including age, tumour size, histological grade, ER status, PR status and HER2 status), proliferation factors (Ki67), and EMT related factors (including Twist and Slug) in the dataset. The cut-off values of ER, PR, HER2 and Ki67 were 1, 1%, 1+/2+, and 14%, respectively, according to the standards of clinical practice. For twist and slug, the final score was 0 to 12 as the cut-off value for the analyses to obtain significant results. Furthermore, multivariate Cox proportional hazard regression analysis was applied to investigate whether the predictive value of the panel was independent of other clinical variables.

The model was established using the and holdout methods, an approach to out-of-sample evaluation, where the dataset was randomly split into two exclusive sets (70% training and 30% testing sets) [43]. The model was then trained on the training group and tested on the testing group 10 times. Additionally, bootstrapping was used to overcome a possible biased error estimate and obtain confidence intervals (CI). We reported the 95% CI of the coefficients, hazard ratio, and relapse rate for each model. Statistical analyses were performed using GraphPad Prism version 6 and R 3.4.0. To enroll more effective biomarkers and clinicopathological factors into further modelling, a *p*-value less than 0.1 was defined as statistically significant in the univariate Cox Proportional Analysis. Then, potential significant factors were enrolled into the multivariate Cox Proportional Analysis, with the *p*-value less than 0.05 considered to be statistically significant. The detail was shown in Additional file 3: Figure S3.

## Results

### Characteristics of patients and IHC results

**Fig. 1:**
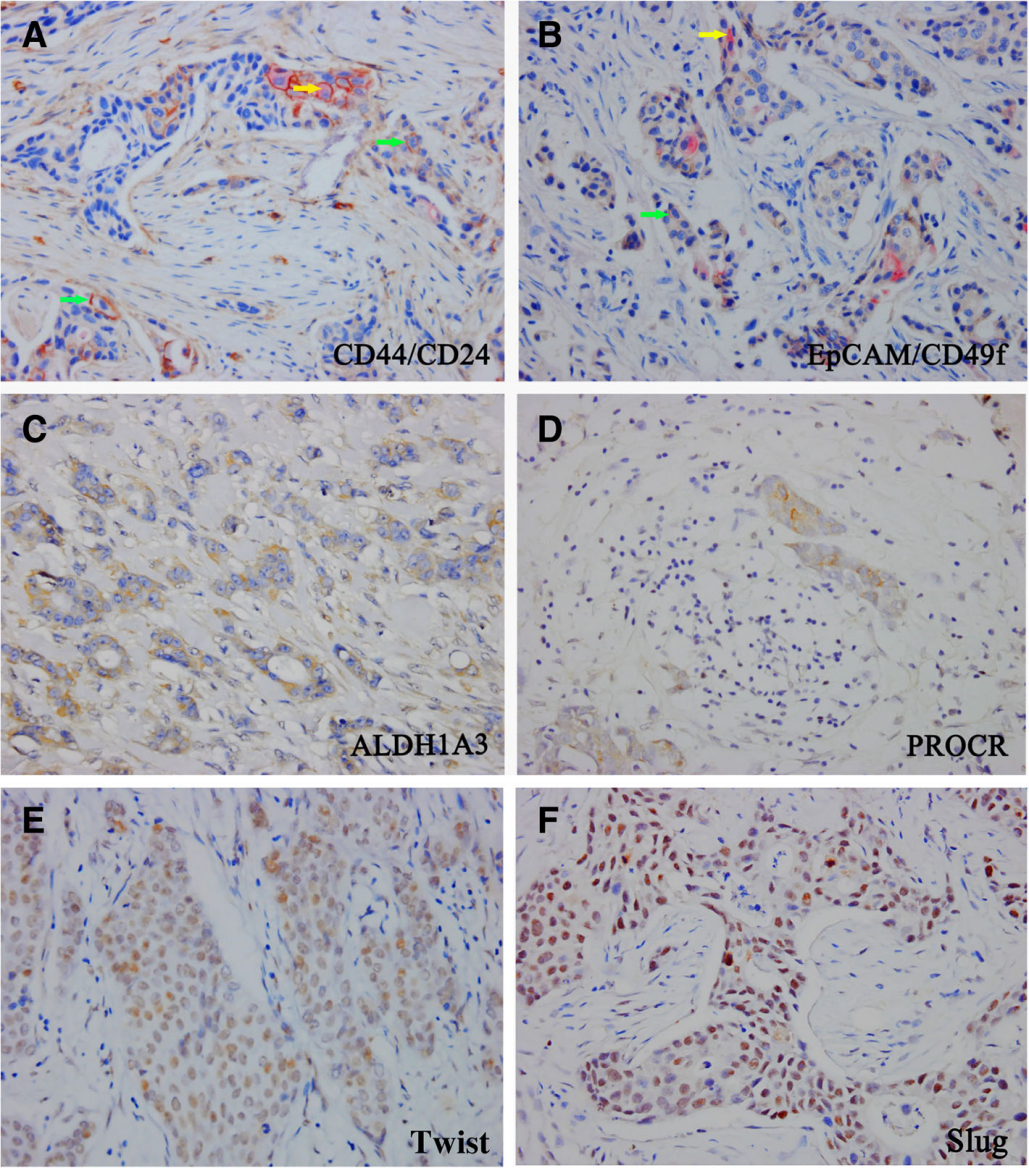
IHC staining in early-stage BIDC patients. **a** Dual staining for CD44 (green arrow) and CD24 (yellow arrow); **b** Dual staining for EpCAM (green arrow) and CD49 (yellow arrow); **c**-**f** Single staining for ALDH1A3 (cytoplasm), PROCR (membrane), Twist (nuclear) and Slug (nuclear), respectively

The mean age of the patients was 49.3 ± 9.9 years. The youngest patient was 23 years old while eldest one was 78 years old. Among the 407 patients, the median follow-up was 66 months, and relapse was observed in 42 (10.3%) patients during five years after diagnosis, consistent with results published in the literature. The characteristics of clinicopathological, proliferation, and EMT related factors of the 407 patients are depicted in Table 1 and Additional file 4: Table S1. IHC staining was performed on slides of paraffin embedded blocks of those 407 BIDC samples. Results are shown in Fig. 1. We also performed IHC in tissues of patients with reductional mammoplasty. The prevalence of BSCCs biomarkers in reductional mammoplasty samples were shown in Additional file 2: Figure S2.

**Table 1:** Characteristics of Clinicopathological, Proliferation, and EMT Related Factors of the 407 Patients

Clinicopathological Factors		Relapse or not(N,%)		*p*-value (log-rank)
		No	Yes	
Age	>40y	307	30 (8.90)	0.016
	≤40y	58	12 (17.15)	
Tumor Size	≤2 cm	165	11 (6.25)	0.032
	> 2 cm	200	31 (13.42)	
Histological Grade	Grade 1	19	0 (0.00)	0.271
	Grade 2	126	18 (12.50)	
	Grade 3	220	24 (9.84)	
ER Status	≤1%(p)	113	12 (9.60)	0.567
	> 1%(n)	252	30 (10.69)	
PR Status	≤1%(p)	135	16 (10.59)	0.722
	> 1%(n)	230	26 (10.16)	
Her2 Status	0/1+	253	31 (10.92)	0.942
	3+	67	7 (9.46)	
Menopausal status	Premenopausal	215	23 (9.66)	0.858
	Postmenopausal	147	15 (9.26)	
Ki67	≤14%	127	11 (7.97)	0.222
	> 14%	238	31 (11.52)	
Twist	TS = 0	175	18 (9.33)	0.560
	TS > 0	190	24 (11.21)	
Slug	TS = 0	231	27 (10.47)	0.722
	TS > 0	134	15 (10.07)	
Surgery	Mastectomy	333	40 (10.72)	0.392
	Lumpectomy	32	2 (5.88)

### Construction and validation of the RRS model

A univariate analysis was performed to test whether the expression level of each BCSC-associated marker was related to differences of patient RFS. Among all the BCSC related biomarkers, four biomarkers (ALDH1A3, CD44^+^/CD24^−^, ITGA6^+^, and PROCR) were confirmed to be statistically correlated with patient RFS (Table 2). The RRS formula according to the expression coefficient of those 4 BCSC-associated biomarkers for survival is listed as follows: RRS = 0.30× (score of ALDH) + 0.34× (score of CD44^+^/CD24^−^) + 0.24× (score of ITGA6) + 0.56× (score of PROCR). Therefore, patients were classified into high-risk and low-risk group individually using the optimal RRS (RRS corresponding to the maximum sum of specificity and sensitivity in the ROC curve) as the cut-off value. With the aid of the method described in the Materials and Methods, the cut-off value was calculated to be 2.05.

**Table 2:** Biomarkers Associated with Relapse in Training Group by Univariate Cox Proportional Analysis

Biomarkers	Coefficient (Wj, 95% CI)	Hazard Radio(95% CI)^a^
ALDH1A3	0.30 (0.27–0.33)	1.35 (1.12–1.58)
CD44^+^/CD24^−^	0.34 (0.31–0.38)	1.41 (1.09–1.72)
ITGA6^+^	0.24 (0.19–0.30)	1.27 (1.04–1.51)
PROCR^+^	0.56 (0.52–0.60)	1.75 (1.49–2.00)

^a^*CI* confidence interval

**Fig. 2:**
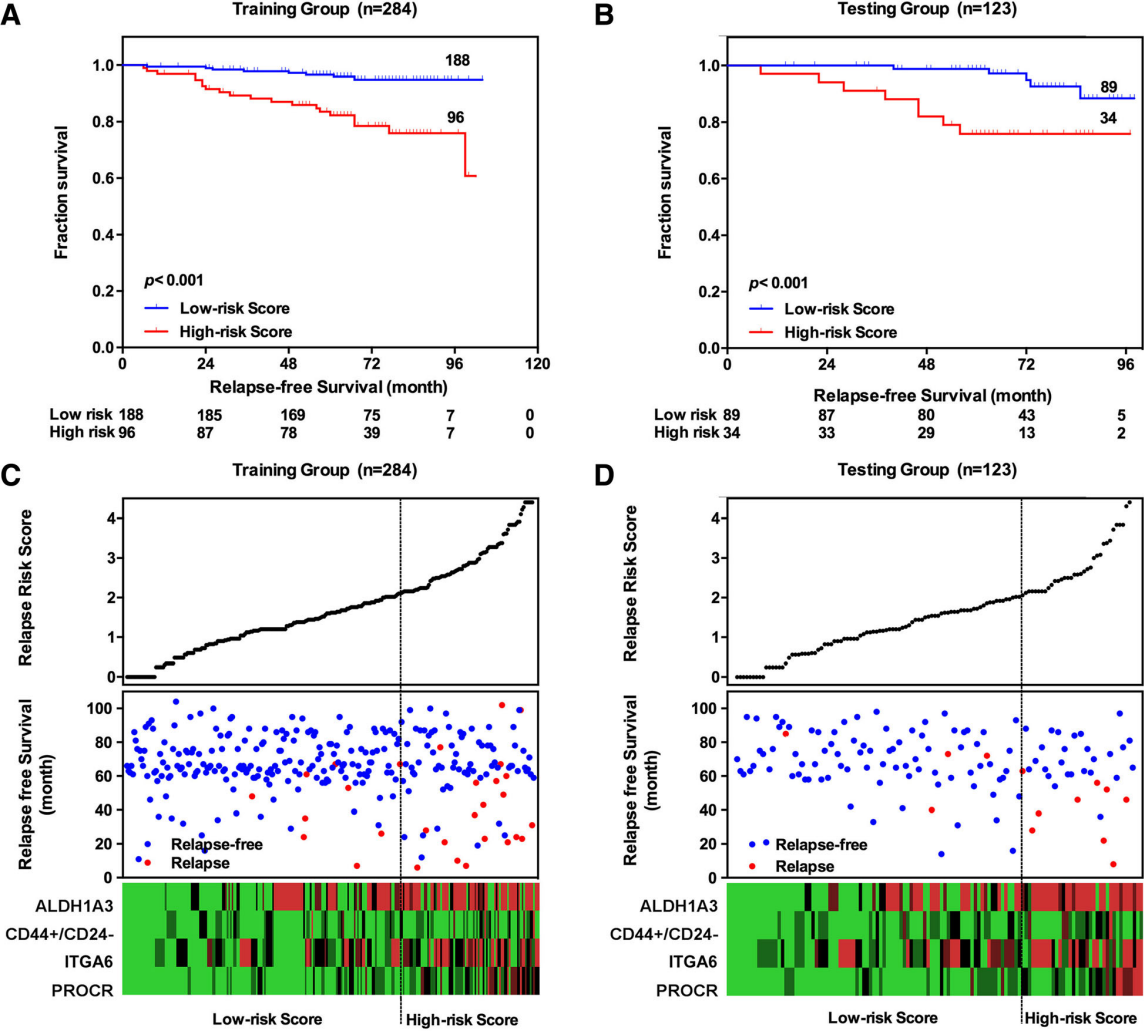
Establishment and Validation of RRS of early-stage BIDC patients, **a** Kaplan-Meier analysis for RFS of early-stage BIDC patients in training group. **b** Kaplan-Meier analysis for RFS of early-stage BIDC patients in testing group. **c** The distribution of the RRS, patients’ relapse status and biomarker expression in training group. **d** The distribution of the RRS, patients’ relapse status and biomarker expression in the testing group. (We conducted 10 times; Fig. 2 is only one example of them)

Then, Kaplan-Meier analysis showed that the proportion of patients in the low-risk group who were free of relapse at 5 years (97.68, 95% CI: 97.37–98.00%) was significantly higher than that in the high-risk group (81.33, 95% CI: 80.50–82.16%) (*p* <  0.001) in the training group. In another exclusive group (the testing group), the proportion of patients in the low-risk group who were free of relapse at 5 years (96.82, 95% CI: 95.88–97.76%) was also higher than that in the high-risk group (82.13, 95% CI: 79.93–84.33%) (p <  0.001). Distributions of risk score, relapse status and BCSC-associated biomarker expression of patients in the training group and testing group is displayed in Table 3 and Fig. 2.

**Table 3:** Kaplan-Meier Estimation of the Rate of Recurrence at 5 Years, According to Recurrence-Score Risk Category

RRS		Percentage of patients (%)	Rate of recurrence at 5 years (%, 95 CI)^a^	*p-*value
Training set	Low-risk	67.54	2.32 (2.00–2.63)	< 0.001
	High-risk	32.46	18.67 (17.84–19.50)	
Testing set	Low-risk	68.46	3.18 (2.24–4.12)	< 0.001
	High-risk	31.54	17.87 (15.67–20.07)	

^a^*CI* confidence interval

Among all the clinicopathological factors (including age at diagnosis, tumour size, histological grade, ER status, PR status and HER2 status), proliferation factors (Ki67), EMT related factors (including Twist and Slug), age at diagnosis and tumour size were considered potential significant factors in the univariate survival analysis. These factors were then fully enrolled to the multivariate Cox model with RRS. In a multiple Cox model, RRS demonstrated significant predictive power that was independent of tumour size and age at diagnosis in both the training group (*p* < 0.001) and testing group (*p* = 0.014) (Table 4).

**Table 4:** Multivariate Cox Proportional Analysis of Tumor Size, age, and RRS in Relation to the Likelihood of Relapse

	*P*-value	Hazard Radio (95% CI) ^a^
Training group		
RRS (high vs. low)	< 0.001	6.75 (2.90–15.72)
Tumor size (> 2 cm vs. ≤2 cm)	0.037	2.72 (1.16–6.38)
Age (>40y vs. ≤40y)	0.098	0.46 (0.20–1.05)
Testing group		
RRS (high vs. low)	0.014	5.04 (1.52–16.81)
Tumor size (> 2 cm vs. ≤2 cm)	0.177	3.33 (0.80–15.85)
Age (>40y vs. ≤40y)	0.316	0.59 (0.15–2.41)

^a^*CI* confidence interval

### Assessment of the RRS model in the entire dataset

#### Assessment of the RRS model in univariate survival analysis (Kaplan-Meier method)

**Fig. 3:**
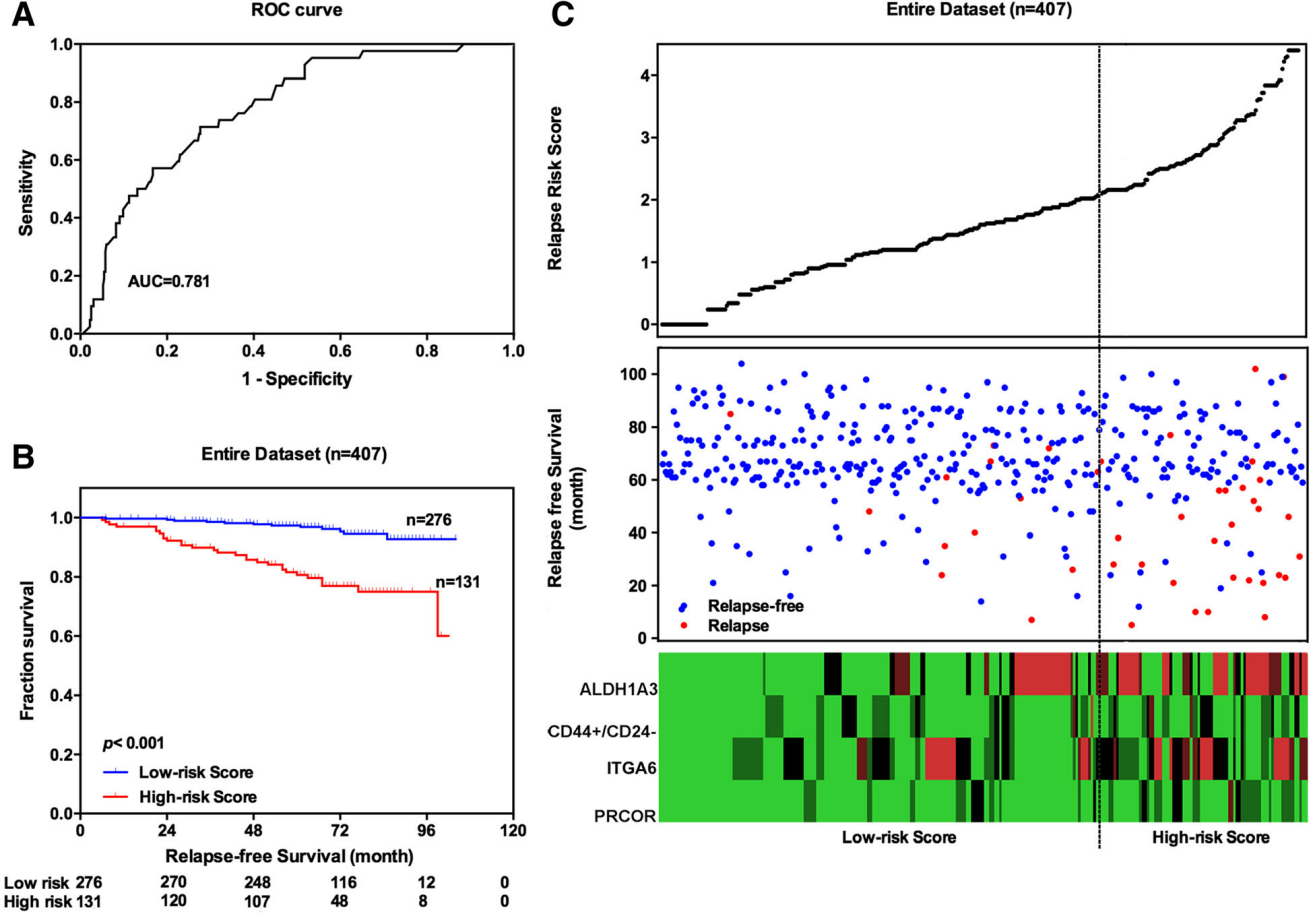
Assessment of RRS of early-stage BIDC patients. **a** The ROC curves for RFS prediction. **b** Kaplan-Meier analysis for RFS of early-stage BIDC patients. **c** The distribution of the RRS, patients’ relapse status and biomarker expression in early-stage BIDC

To validate our findings, the RRS model was assessed in the entire dataset (*n* = 407). By using the same cut-off value of training groups, patients in the entire dataset were classified into the high-risk group (*n* = 131) and low-risk group (*n* = 276) (Fig. 3a). Patients with high risk scores demonstrated significantly reduced RFS when compared to those with low risk scores (log-rank test *p* < 0.001) (Fig. 3b). The relapse rate at 5 years was 19.30% (95% CI: 12.34–26.27%) and 2.67% (95% CI: 0.72–4.63%) in the high-risk group and low-risk group, respectively. Distributions of risk score, relapse status and BCSC-associated biomarker expression of each patient in the entire datasets were then analysed (Fig. 3c).

#### Assessment of the RRS model in multivariate survival analysis (cox proportional analysis)

In the entire dataset, the correlation between RFS and clinicopathological factors (including age, tumour size, histological grade, ER status, PR status and HER2 status), proliferation factors (Ki67), EMT related factors (including Twist and Slug) was analysed by *Kaplan-Meier* method. Reduced RFS was only demonstrated in patients with smaller tumour size (log-rank *p* = 0.032) and younger age (log-rank *p* = 0.016) (Table 1). Then, multivariate survival analyses were adopted to explore the association between relapse and age as well as tumour size. As a result, younger age, larger tumour size and RRS were implied to be significant predictors of relapse (Table 5).

**Table 5:** Multivariate Cox Proportional Analysis of Age, Tumor Size, and RRS in Relation to the Likelihood of Relapse in Entire Dataset

Variable	*p*-value	Hazard Ratio (95%CI)^a^
Analysis without RRS		
Age (≤40y vs. >40y)	0.012	2.38 (1.21–4.69)
Tumor Size (> 2 cm vs. ≤2 cm)	0.022	2.22 (1.11–4.44)
Analysis with RRS		
Age (≤40y vs. >40y)	0.022	2.22 (1.12–4.39)
Tumor Size (> 2 cm vs. ≤2 cm)	0.005	2.70 (1.34–5.41)
RRS (high vs. low)	< 0.001	5.92 (3.01–11.6)

^a^*CI* confidence interval

#### Hormone therapy benefit in different groups

**Fig. 4:**
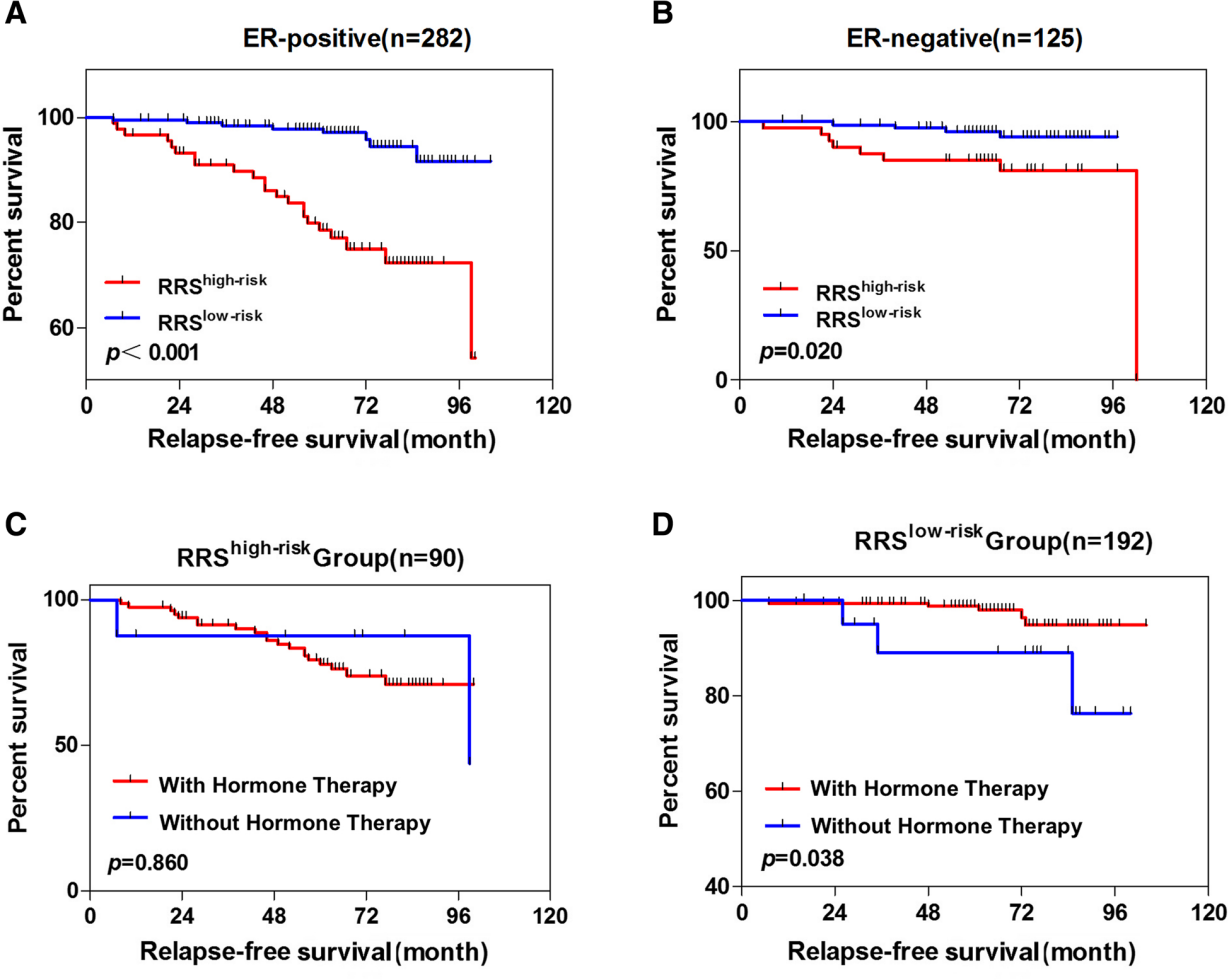
Kaplan-Meier analysis for RFS using RRS in the subgroups stratified by ER status and endocrine therapy. **a** Kaplan-Meier curves for early-stage BIDC patients with ER-positive status. **b** Kaplan-Meier curves for early-stage BIDC patients with ER-negative status. **c** Kaplan-Meier curves for ER-positive patients with high risk scores stratified by endocrinotherapy. **d** Kaplan-Meier curves for ER-positive patients with low risk scores stratified by endocrinotherapy

Among the 407 patients, there were 282 ER-positive and 125 ER-negative patients. We found that our panel worked in both of these two subgroups (Fig. 4a, b). In the ER-positive group, all patients were treated with chemotherapy, whereas only 89.72% (*n* = 253) of these patients received hormone therapy. Our results demonstrated no difference for the RFS between those hormone-treated patients and non-treated patients in the high-risk score group (*p* = 0.860 Fig. 4d). However, in the low-risk score group, patients in the treated group showed remarkably longer RFS than those in the non-treated group (*p* = 0.038, Fig. 4c), which indicated that patients with a high-risk score may not benefit from the traditional hormone therapy.

## Discussion

An increasing number of females are diagnosed with node negative invasive breast carcinomas. Even though most of patients with early-stage breast cancer have a favourable outcome, the 5-year rate of local relapse or distant metastasis in our dataset is still up to 10.3%. As metastatic diseases are challenging to cure, accurate evaluation for prognosis and more efficacious treatments are needed. In our present study, we developed and validated a novel prognostic model based on 4 BCSC-associated biomarkers to improve our accuracy of predicting disease recurrence in patients with early stage BIDC (T_1–3_N_0_M_0_). The four biomarkers incorporated into our predictive model have been shown to be involved in stem cell ability in vivo and in vitro, including self-renewal ability and tumorigenic capacity, which could contribute greatly to metastasis of BIDC in vitro and in vivo, or in tumour tissues [21–25, 44–46].

The holdout methods were adopted to establish our RRS model, which assisted us to obtain a stable model to calculate RRS in our study. Our model was further validated in the entire dataset. The AUC value of ROC curve is 0.781 which indicated that the RRS is a good classifier for relapse among patients with early stage breast cancer. The difference in the risk of relapse between patients with low risk scores and those with high-risk scores was large and statistically significant. There are 276 (67.81%) patients who were classified in the low-risk group, while only 32.19% of patients were included in the high-risk group, and their rate of relapse at 5 years was 19.30 and 2.67%, respectively. Therefore, the application of the RRS predictor provides a good estimate of the risk of local or distant recurrence in individual patients.

We also enrolled other biomarkers in the univariate survival analysis in the training set, such as age, tumour size, histological grade, Ki67, and EMT related biomarkers. All those parameters have been reported to play critical roles in accelerating the presence of distant metastasis or local relapse [47, 48]. Despite the fact that EMT has been reported to produce cells with stem cell-like properties [49], we found that no parameter showed significantly different RFS in different subgroups of EMT related biomarkers. In this study, smaller tumour size was validated as an independent factor protecting patients from relapse. When the RRS was combined with data pertaining to tumour size to predict the risk of relapse, the relapse score remained statistically significant in a multivariate analysis.

Due to poor compliance of our patients, in the ER-positive subgroups, only 89.72% of patients received endocrine therapy systematically. The results indicated that only patients with low risk responded well to endocrine therapy, while those with high risk showed no difference between the treated group and untreated group. A previous study revealed that mesenchymal-like BCSCs in hormone-sensitive luminal breast cancers were one of the reasons for hormone-resistant [50]. Similar to above finding, there was evidence suggesting that BCSCs should be partially responsible for the endocrine-resistant capacity of breast cancer cells. This is due to the fact that CSCs could only respond to treatment by virtue of paracrine signalling pathway from adjacent differentiated ER-positive tumour cells [51–54], which were probably responsible for the endocrine-resistance in the high-risk group.

The RRS not only offers an approach to predict therapeutic sensitivity but also provides a new perspective to eliminate BCSCs in early stage breast cancer. As been reported, BCSCs were not as sensitive to hormone therapy and conventional chemotherapy as non-BCSC tumours. Thus, targeting BCSCs clinically might enhance the therapeutic sensitivity among patients with high risk scores. The most promising CSC treatment strategies that target Notch, Hedgehog, Wnt and many other BCSC self-renewal pathways provide a number of opportunities for new clinical trials.^20^ In addition, the strategy of “destemming” CSCs, including inducing CSC differentiation or inhibiting self-renewal capacity were also recommended [55]. Combination of BCSC-targeted therapy and traditional therapy may provide our patients with high-risk scores more effective therapeutic strategies. However, the study of CSCs remains an enigma, and further exploration is needed.

In terms of limitations, this study was a retrospective analysis that selected patients who had not received neoadjuvant chemotherapy after resection in early stage breast cancer, which may lead to a selection bias of patients with a relative lower risk of recurrence. However, all our patients included in this study were T_1–3_N_0_M_0_ by the TNM staging system, and the majority of them did not receive neoadjuvant chemotherapy, according to the NCCN guideline [12]. The total study size is modest in absolute numbers, and some subgroup analyses may be underpowered; however, this is one of the largest cohorts of well-characterized early stage breast cancer that employed a BCSC biomarker panel as a prognosis model. The shortcomings of this panel should not be ignored. First of all, though IHC staining is the most common method for semi-quantified the protein expression level in carcinomous tissues, the subjectivity of evaluation of this method couldn’t be avoided. Secondly, the selection of antibodies should be cautiously considered, as their quality will affect the result of IHC staining directly. Performing immunofluorescence staining and q-RT PCR may help us obtain a relative exact result; however, these two methods also have their disadvantages in assessing BCSCs.

## Conclusion

Though previous studies have combined different BCSCs biomarkers for assessing prognosis in different types of breast cancer, such as three-negative, HER2-positive and metastatic breast cancer [56–59], no BCSC-associated biomarkers have been combined to form a model for evaluating the relapse risk of early-stage breast cancer. We propose that BCSCs could be used as a panel in prognostic or predictive tests of early-stage breast cancer. Here, we conducted a prospectively designed validation study of a multi-biomarker panel in a cohort of patients with early-stage BIDC. In addition, this panel is promising for prediction of early-stage BIDC recurrence, the efficacy of which warrants further validation in a large-scale cohort. In addition, it reminds us that further consideration is needed to explore new therapeutic managements for high-risk patients with therapeutic resistance. In addition, it is of practical significance that the panel only involves the use of routine slides of the tumour tissues and five antibodies, which is not as time-consuming and expensive as other gene profiles.

## Additional files

Additional file 1: **Figure S1.** Different expression patterns of BSCCs biomarkers expression pattern in external control and internal control tissues. A. ALDH1A3 was shown positive in prostate cancer (external control) and breast invasive ductal carcinoma (IDC, internal positive control), and shown negative in lymphocytes (internal negative control); B. PROCR was shown positive in intestine gland (external control) and ductal carcinoma in situ (DCIS, internal positive control), and shown negative in lymphocytes (internal negative control); C. CD44 was shown positive in urothelium (external control) and IDC (internal positive control), and shown negative in lymphocytes (internal negative control); D. CD24 was shown positive in urothelium (external control) and IDC (internal positive control), and shown negative in breast adenosis (internal negative control); E. EpCAM was shown positive in intestine gland (external control) and in breast adenosis (internal positive control), and shown negative in lymphocytes (internal negative control); F. ITGA6 was shown positive in colorectal carcinoma (external control) and in IDC (internal positive control), and shown negative in lymphocytes (internal negative control). (JPG 5319 kb)

Additional file 2: **Figure S2.** The prevalence of BSCCs biomarkers in reductional mammoplasty samples. A. Prevalence of ALDH1A3 in three in reductional mammoplasty samples; B. Prevalence of PROCR in three in reductional mammoplasty samples; C-D. Prevalence of CD44/CD24 in three in reductional mammoplasty samples; E. Prevalence of EpCAM in three in reductional mammoplasty samples; F. Prevalence of ITGA6 in three in reductional mammoplasty samples. (JPG 4739 kb)

Additional file 3: **Figure S3.** Flow Chart for Construction of RRS model. (JPG 293 kb)

Additional file 4: **Table S1.** The detailed information of end-point of follow-up for local recurrence or distant metastasis. (XLSX 124 kb)

Additional file 5: **Table S2.** Antibodies used in the cohort of patients. (DOCX 16 kb)

## Data Availability

All data generated or analysed during this study are included in this published article and its supplementary information files.

## Abbreviations

BCSCs: Breast cancer stem cells
BIDC: Breast invasive ductal carcinoma
CI: Confidence intervals
CSC: Cancer stem cell
EMT: Epithelial-to-mesenchymal transition
ER: Oestrogen receptor
GEF: Gene expression profiling
H&E: Haematoxylin and eosin
HER2: Human epidermal growth factor receptor 2
IHC: Immunohistochemistry
PR: Progesterone receptor
RFS: Relapse-free survival
ROC: Receiver operating characteristic curve
RRS: Relapse risk score

## References

[1] Iqbal J, Ginsburg O, Rochon PA, Sun P, Narod SA (2015). Differences in breast cancer stage at diagnosis and cancer-specific survival by race and ethnicity in the United States. JAMA..

[2] Kent C, Horton J, Blitzblau R, Koontz BF (2015). Whose disease will recur after mastectomy for early stage, node-negative breast cancer? A systematic review. Clin Breast Cancer.

[3] Verma A, Kaur J, Mehta K (2015). Molecular oncology update: breast cancer gene expression profiling. Asian J Oncol.

[4] Buyse M, Loi S, van't Veer L, Viale G, Delorenzi M, Glas AM, d'Assignies MS, Bergh J, Lidereau R, Ellis P, Harris A, Bogaerts J, Therasse P, Floore A, Amakrane M, Piette F, Rutgers E, Sotiriou C, Cardoso F, Piccart MJ (2006). TRANSBIG consortium. Validation and clinical utility of a 70-gene prognostic signature for women with node-negative breast cancer. J Natl Cancer Inst.

[5] Mook S, Schmidt MK, Viale G, Pruneri G, Eekhout I, Floore A, Glas AM, Bogaerts J, Cardoso F, Piccart-Gebhart MJ, Rutgers ET, Van't Veer LJ (2009). TRANSBIG consortium. The 70-gene prognosis-signature predicts disease outcome in breast cancer patients with 1-3 positive lymph nodes in an independent validation study. Breast Cancer Res Treat.

[6] Gnant M, Sestak I, Filipits M, Dowsett M, Balic M, Lopez-Knowles E, Greil R, Dubsky P, Stoeger H, Rudas M, Jakesz R, Ferree S, Cowens JW, Nielsen T, Schaper C, Fesl C, Cuzick J (2015). Identifying clinically relevant prognostic subgroups of postmenopausal women with node-positive hormone receptor-positive early-stage breast cancer treated with endocrine therapy: a combined analysis of ABCSG-8 and ATAC using the PAM50 risk of recurrence score and intrinsic subtype. Ann Oncol.

[7] Paik S, Shak S, Tang G, Kim C, Baker J, Cronin M, Baehner FL, Walker MG, Watson D, Park T, Hiller W, Fisher ER, Wickerham DL, Bryant J, Wolmark N (2004). A multigene assay to predict recurrence of tamoxifen-treated, node-negative breast cancer. N Engl J Med.

[8] Paik S, Tang G, Shak S, Kim C, Baker J, Kim W, Cronin M, Baehner FL, Watson D, Bryant J, Costantino JP, Geyer CE, Wickerham DL, Wolmark N (2006). Gene expression and benefit of chemotherapy in women with node-negative, estrogen receptor-positive breast cancer. J Clin Oncol.

[9] Foekens JA, Atkins D, Zhang Y, Sweep FC, Harbeck N, Paradiso A, Cufer T, Sieuwerts AM, Talantov D, Span PN, Tjan-Heijnen VC, Zito AF, Specht K, Hoefler H, Golouh R, Schittulli F, Schmitt M, Beex LV, Klijn JG, Wang Y (2006). Multicenter validation of a gene expression-based prognostic signature in lymph node-negative primary breast cancer. J Clin Oncol.

[10] Sotiriou C, Wirapati P, Loi S, Harris A, Fox S, Smeds J, Nordgren H, Farmer P, Praz V, Haibe-Kains B, Desmedt C, Larsimont D, Cardoso F, Peterse H, Nuyten D, Buyse M, Van de Vijver MJ, Bergh J, Piccart M, Delorenzi M (2006). Gene expression profiling in breast cancer: understanding the molecular basis of histologic grade to improve prognosis. J Natl Cancer Inst.

[11] Filipits M, Rudas M, Jakesz R, Dubsky P, Fitzal F, Singer CF, Dietze O, Greil R, Jelen A, Sevelda P, Freibauer C, Müller V, Jänicke F, Schmidt M, Kölbl H, Rody A, Kaufmann M, Schroth W, Brauch H, Schwab M, Fritz P, Weber KE, Feder IS, Hennig G, Kronenwett R, Gehrmann M, Gnant M (2011). A new molecular predictor of distant recurrence in ER-positive, HER2-negative breast cancer adds independent information to conventional clinical risk factors. Clin Cancer Res.

[12] National Comprehensive Cancer Network: Practice Guidelines in Oncology. Invasive Breast Cancer, version 3. 2018. https://www.nccn.org/patients/guidelines/breast-invasive/.

[13] Guo W (2014). Concise review: breast cancer stem cells: regulatory networks, stem cell niches, and disease relevance. Stem Cells Transl Med.

[14] Al-Hajj M, Wicha MS, Benito-Hernandez A, Morrison SJ, Clarke MF (2003). Prospective identification of tumorigenic breast cancer cells. Proc Natl Acad Sci U S A.

[15] Geng SQ, Alexandrou AT, Li JJ (2014). Breast cancer stem cells: multiple capacities in tumor metastasis. Cancer Lett.

[16] Bozorgi A, Khazaei M, Khazaei MR (2015). New findings on breast cancer stem cells: a review. J Breast Cancer.

[17] Smalley M, Piggott L, Clarkson R (2013). Breast cancer stem cells: obstacles to therapy. Cancer Lett.

[18] Korkaya H, Wicha MS (2013). HER2 and breast cancer stem cells: more than meets the eye. Cancer Res.

[19] Reya T, Morrison SJ, Clarke MF, Weissman IL (2001). Stem cells, cancer, and cancer stem cells. Nature..

[20] Singh SK, Hawkins C, Clarke ID, Squire JA, Bayani J, Hide T, Henkelman RM, Cusimano MD, Dirks PB (2004). Identification of human brain tumour initiating cells. Nature..

[21] Ginestier C, Hur MH, Charafe-Jauffret E, Monville F, Dutcher J, Brown M, Jacquemier J, Viens P, Kleer CG, Liu S, Schott A, Hayes D, Birnbaum D, Wicha MS, Dontu G (2007). ALDH1 is a marker of normal and malignant human mammary stem cells and a predictor of poor clinical outcome. Cell Stem Cell.

[22] Mannello F (2013). Understanding breast cancer stem cell heterogeneity: time to move on to a new research paradigm. BMC Med.

[23] Luo M, Clouthier SG, Deol Y, Liu S, Nagrath S, Azizi E, Wicha MS (2015). Breast cancer stem cells: current advances and clinical implications. Methods Mol Biol.

[24] Wang D, Cai C, Dong X, Yu QC, Zhang XO, Yang L, Zeng YA (2015). Identification of multipotent mammary stem cells by protein C receptor expression. Nature..

[25] Iqbal J, Chong PY, Tan PH (2013). Breast cancer stem cells: an update. J Clin Pathol.

[26] Oakes SR, Gallego-Ortega D, Ormandy CJ (2014). The mammary cellular hierarchy and breast cancer. Cell Mol Life Sci.

[27] Abraham BK, Fritz P, McClellan M, Hauptvogel P, Athelogou M, Brauch H (2005). Prevalence of CD44+/CD24−/low cells in breast cancer may not be associated with clinical outcome but may favor distant metastasis. Clin Cancer Res.

[28] Ali HR, Dawson SJ, Blows FM, Provenzano E, Pharoah PD, Caldas C (2011). Cancer stem cell markers in breast cancer: pathological, clinical and prognostic significance. Breast Cancer Res.

[29] Honeth G, Schiavinotto T, Vaggi F, Marlow R, Kanno T, Shinomiya I, Lombardi S, Buchupalli B, Graham R, Gazinska P, Ramalingam V, Burchell J, Purushotham AD, Pinder SE, Csikasz-Nagy A, Dontu G (2015). Models of breast morphogenesis based on localization of stem cells in the developing mammary lobule. Stem Cell Rep.

[30] Goel HL, Gritsko T, Pursell B, Chang C, Shultz LD, Greiner DL, Norum JH, Toftgard R, Shaw LM, Mercurio AM (2014). Regulated splicing of the α6 integrin cytoplasmic domain determines the fate of breast cancer stem cells. Cell Rep.

[31] Lim E, Vaillant F, Wu D, Forrest NC, Pal B, Hart AH, Asselin-Labat ML, Gyorki DE, Ward T, Partanen A, Feleppa F, Huschtscha LI, Thorne HJ, Fox SB, Yan M, French JD, Brown MA, Smyth GK, Visvader JE, Lindeman GJ, kConFab (2009). Aberrant luminal progenitors as the candidate target population for basal tumor development in BRCA1 mutation carriers. Nat Med.

[32] Turner NC, Reis-Filho JS (2006). Basal-like breast cancer and the BRCA1 phenotype. Oncogene..

[33] Beaulieu LM, Church FC (2007). Activated protein C promotes breast cancer cell migration through interactions with EPCR and PAR-1. Exp Cell Res.

[34] Spek CA, Arruda VR (2012). The protein C pathway in cancer metastasis. Thromb Res.

[35] Qiu Y, Pu T, Li L, Cheng F, Lu C, Sun L, Teng X, Ye F, Bu H (2014). The expression of aldehyde dehydrogenase family in breast cancer. J Breast Cancer.

[36] Yan Q, Zhong X, Zhang Z, Bing W, Feng Y, Hong B (2017). Prevalence of protein C receptor (PROCR) is associated with inferior clinical outcome in breast invasive ductal carcinoma. Pathol Res Pract.

[37] Ye F, Zhong X, Qiu Y, Yang L, Wei B, Zhang Z, Bu H (2017). ITGA6 can act as a biomarker for local or distant recurrence in breast cancer. J Breast Cancer.

[38] Wolff AC, Hammond ME, Hicks DG, Dowsett M, LM MS, Allison KH, Allred DC, Bartlett JM, Bilous M, Fitzgibbons P, Hanna W, Jenkins RB, Mangu PB, Paik S, Perez EA, Press MF, Spears PA, Vance GH, Viale G, Hayes DF, American Society of Clinical Oncology; College of American Pathologists (2014). Recommendations for human epidermal growth factor receptor 2 testing in breast cancer: American Society of Clinical Oncology/College of American Pathologists clinical practice guideline update. Arch Pathol Lab Med.

[39] Qureshi A, Pervez S (2010). Allred scoring for ER reporting and it's impact in clearly distinguishing ER negative from ER positive breast cancers. J Pak Med Assoc.

[40] Spizzo G, Obrist P, Ensinger C, Theurl I, Dünser M, Ramoni A, Gunsilius E, Eibl G, Mikuz G, Gastl G (2002). Prognostic significance of ep-CAM AND Her-2/neu overexpression in invasive breast cancer. Int J Cancer.

[41] Sun J, Chen X, Wang Z, Guo M, Shi H, Wang X, Cheng L, Zhou M (2015). A potential prognostic long non-coding RNA signature to predict metastasis-free survival of breast cancer patients. Sci Rep.

[42] Hu Z, Chen X, Zhao Y, Tian T, Jin G, Shu Y, Chen Y, Xu L, Zen K, Zhang C, Shen H (2010). Serum microRNA signatures identified in a genome-wide serum microRNA expression profiling predict survival of non-small-cell lung cancer. J Clin Oncol.

[43] Mohebian MR, Marateb HR, Mansourian M, Mañanas MA, Mokarian FA (2017). A hybrid computer-aided-diagnosis system for prediction of breast cancer recurrence (HPBCR) using optimized ensemble learning. Comput Struct Biotechnol J.

[44] Sheridan C, Kishimoto H, Fuchs RK, Mehrotra S, Bhat-Nakshatri P, Turner CH, Goulet R, Badve S, Nakshatri H (2006). CD44+/CD24- breast cancer cells exhibit enhanced invasive properties: an early step necessary for metastasis. Breast Cancer Res.

[45] Ye F, Qiu Y, Li L, Yang L, Cheng F, Zhang H, Wei B, Zhang Z, Sun L, Bu H (2015). The presence of EpCAM(−)/ITGA6(+) cells in breast cancer is associated with a poor clinical outcome. J Breast Cancer.

[46] Ghebeh H, Sleiman GM, Manogaran PS, Al-Mazrou A, Barhoush E, Al-Mohanna FH, Tulbah A, Al-Faqeeh K, Adra CN (2013). Profiling of normal and malignant breast tissue show CD44high/CD24low phenotype as a predominant stem/progenitor marker when used in combination with ep-CAM/ITGA6 markers. BMC Cancer.

[47] Cianfrocca M, Goldstein LJ (2004). Prognostic and predictive factors in early-stage breast cancer. Oncologist..

[48] Bill R, Christofori G (2015). The relevance of EMT in breast cancer metastasis: correlation or causality?. FEBS Lett.

[49] Mallini P, Lennard T, Kirby J, Meeson A (2014). Epithelial-to-mesenchymal transition: what is the impact on breast cancer stem cells and drug resistance. Cancer Treat Rev.

[50] Creighton CJ, Li X, Landis M, Dixon JM, Neumeister VM, Sjolund A, Rimm DL, Wong H, Rodriguez A, Herschkowitz JI, Fan C, Zhang X, He X, Pavlick A, Gutierrez MC, Renshaw L, Larionov AA, Faratian D, Hilsenbeck SG, Perou CM, Lewis MT, Rosen JM, Chang JC (2009). Residual breast cancers after conventional therapy display mesenchymal as well as tumor-initiating features. Proc Natl Acad Sci U S A.

[51] O'Brien CS, Howell SJ, Farnie G, Clarke RB (2009). Resistance to endocrine therapy: are breast cancer stem cells the culprits?. J Mammary Gland Biol Neoplasia.

[52] O'Brien CS, Farnie G, Howell SJ, Clarke RB (2011). Breast cancer stem cells and their role in resistance to endocrine therapy. Horm Cancer.

[53] Stone A, Musgrove EA (2014). Endocrine therapy: defining the path of least resistance. Breast Cancer Res.

[54] Arif K, Hussain I, Rea C, El-Sheemy M (2015). The role of Nanog expression in tamoxifen-resistant breast cancer cells. Onco Targets Ther.

[55] Wang T, Shigdar S, Gantier MP, Hou Y, Wang L, Li Y, Shamaileh HA, Yin W, Zhou SF, Zhao X, Duan W (2015). Cancer stem cell targeted therapy: progress amid controversies. Oncotarget..

[56] Yang F, Cao L, Sun Z, Jin J, Fang H, Zhang W, Guan X (2016). Evaluation of breast Cancer stem cells and Intratumor Stemness heterogeneity in triple-negative breast Cancer as prognostic factors. Int J Biol Sci.

[57] Seo AN, Lee HJ, Kim EJ, Jang MH, Kim YJ, Kim JH, Kim SW, Ryu HS, Park IA, Im SA, Gong G, Jung KH, Kim HJ, Park SY (2016). Expression of breast cancer stem cell markers as predictors of prognosis and response to trastuzumab in HER2-positive breast cancer. Br J Cancer.

[58] Oon ML, Thike AA, Tan SY, Tan PH (2015). Cancer stem cell and epithelial-mesenchymal transition markers predict worse outcome in metaplastic carcinoma of the breast. Breast Cancer Res Treat.

[59] Giordano A, Gao H, Anfossi S, Cohen E, Mego M, Lee BN, Tin S, De Laurentiis M, Parker CA, Alvarez RH, Valero V, Ueno NT, De Placido S, Mani SA, Esteva FJ, Cristofanilli M, Reuben JM (2012). Epithelial-mesenchymal transition and stem cell markers in patients with HER2-positive metastatic breast cancer. Mol Cancer Ther.

