# Supplementary figures and images for: A multiple breast cancer stem cell model to predict recurrence of T1–3, N0 breast cancer
Source: BMC Cancer. 2019 Jul 24;19:729. doi: 10.1186/s12885-019-5941-5 (PMC6657050; doi:10.1186/s12885-019-5941-5)

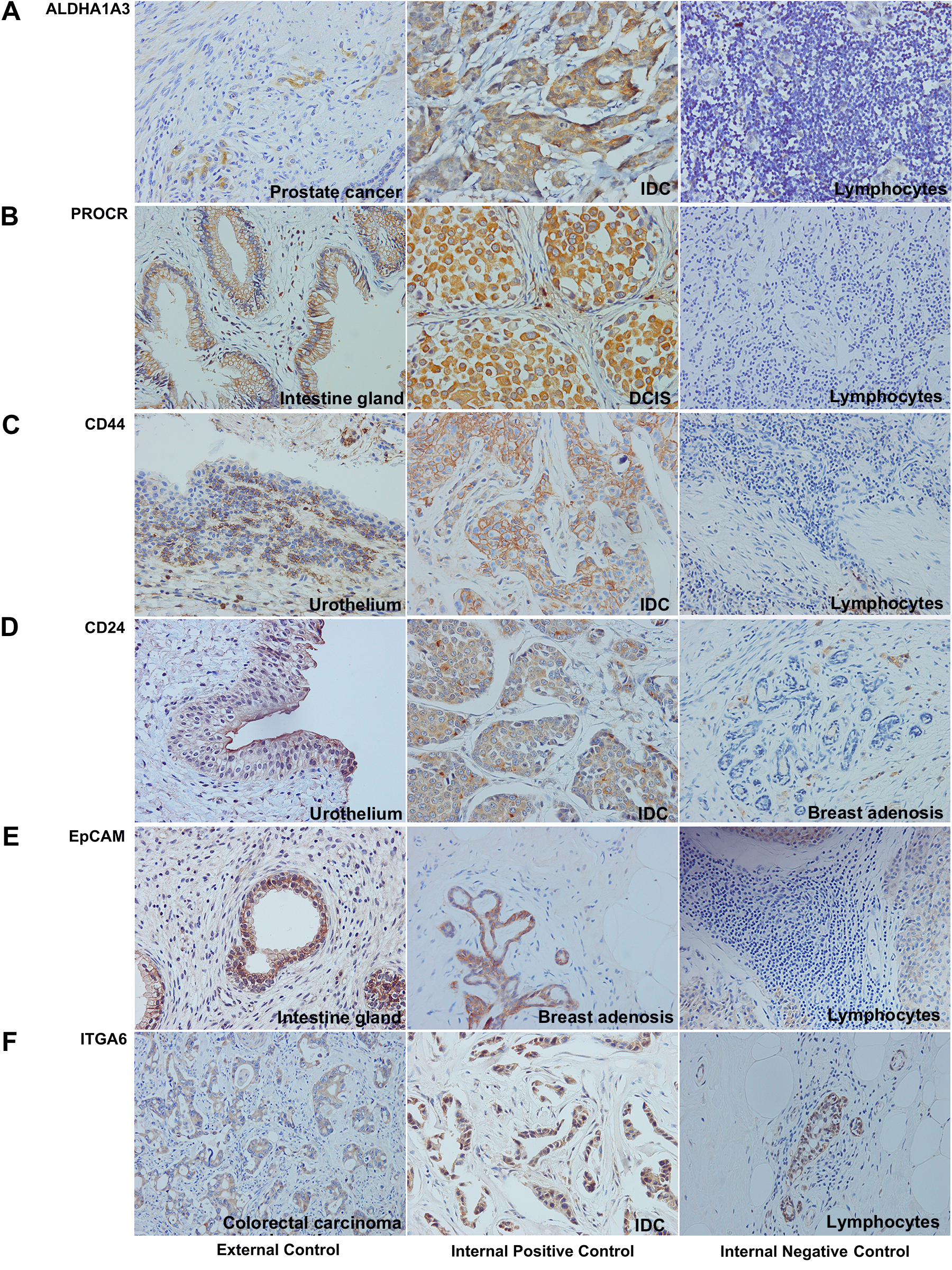

Supplement: Supplementary file 1 — Figure S1. Different expression patterns of BSCCs biomarkers expression pattern in external control and internal control tissues. A. ALDH1A3 was shown positive in prostate cancer (external control) and breast invasive ductal carcinoma (IDC, internal positive control), and shown negative in lymphocytes (internal negative control); B. PROCR was shown positive in intestine gland (external control) and ductal carcinoma in situ (DCIS, internal positive control), and shown negative in lymphocytes (internal negative control); C. CD44 was shown positive in urothelium (external control) and IDC (internal positive control), and shown negative in lymphocytes (internal negative control); D. CD24 was shown positive in urothelium (external control) and IDC (internal positive control), and shown negative in breast adenosis (internal negative control); E. EpCAM was shown positive in intestine gland (external control) and in breast adenosis (internal positive control), and shown negative in lymphocytes (internal negative control); F. ITGA6 was shown positive in colorectal carcinoma (external control) and in IDC (internal positive control), and shown negative in lymphocytes (internal negative control). (JPG 5319 kb) [file 12885_2019_5941_MOESM1_ESM.jpg]

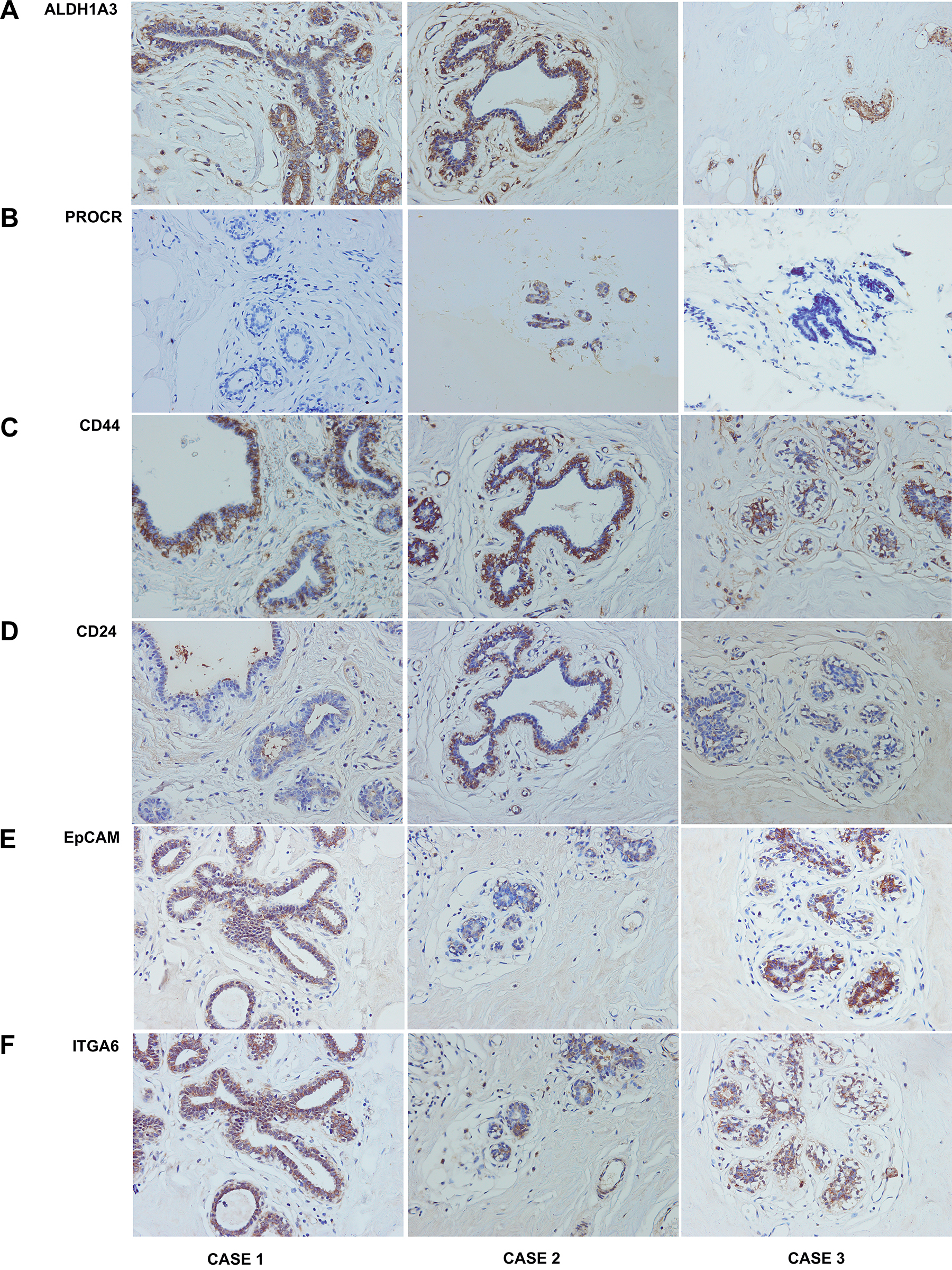

Supplement: Supplementary file 2 — Figure S2. The prevalence of BSCCs biomarkers in reductional mammoplasty samples. A. Prevalence of ALDH1A3 in three in reductional mammoplasty samples; B. Prevalence of PROCR in three in reductional mammoplasty samples; C-D. Prevalence of CD44/CD24 in three in reductional mammoplasty samples; E. Prevalence of EpCAM in three in reductional mammoplasty samples; F. Prevalence of ITGA6 in three in reductional mammoplasty samples. (JPG 4739 kb) [file 12885_2019_5941_MOESM2_ESM.jpg]

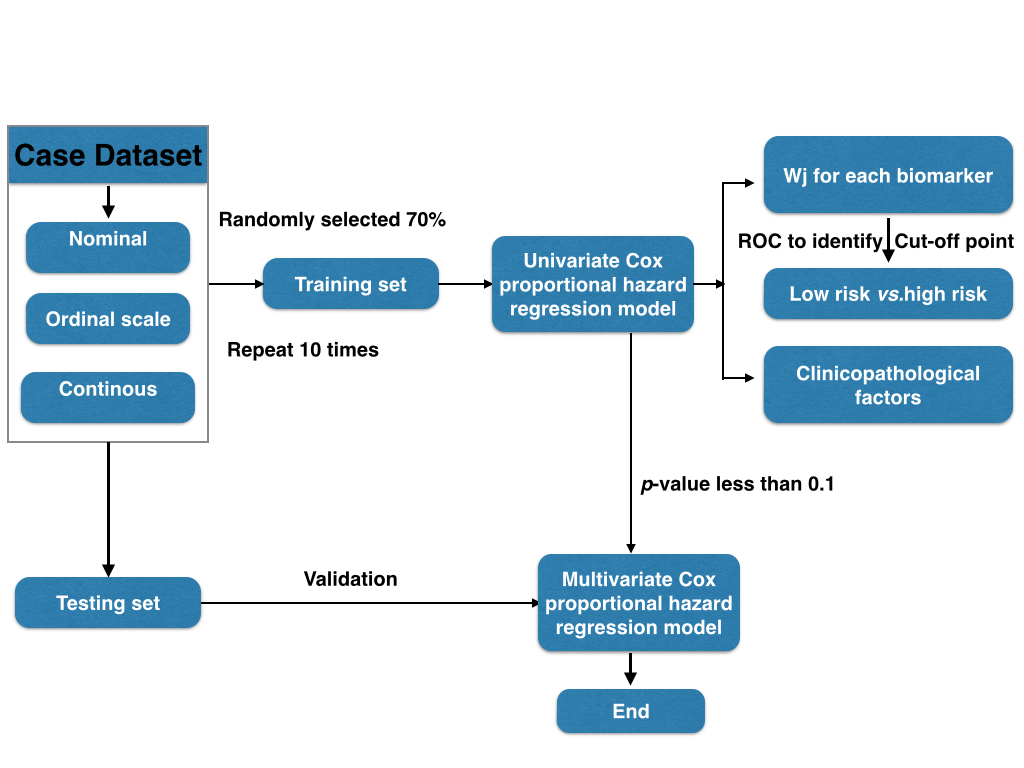

Supplement: Supplementary file 3 — Figure S3. Flow Chart for Construction of RRS model. (JPG 293 kb) [file 12885_2019_5941_MOESM3_ESM.jpg]
