# Supplementary material for: A multiple breast cancer stem cell model to predict recurrence of T1–3, N0 breast cancer
Source: BMC Cancer. 2019 Jul 24;19:729. doi: 10.1186/s12885-019-5941-5 (PMC6657050; doi:10.1186/s12885-019-5941-5)
Supplement: Supplementary file 5 — Table S2. Antibodies used in the cohort of patients. (DOCX 16 kb) [file 12885_2019_5941_MOESM5_ESM.docx]

| **Table S2. Antibodies used in the cohort of patients** | | | | |
| --- | --- | --- | --- | --- |
| **Antibodies** | **Company** | **Clone No.** | **Origin** | **Dilution** |
| **CD44** | Abcam | Ab51037 | Rabbit | 1:50 |
| **CD24** | Merck Millipore | CBL561 | Mouse | 1:1200 |
| **ALDH1A3** | Origene | TA502805 | Mouse | 1:400 |
| **CD49f** | Origene | TA310957 | Mouse | 1:300 |
| **EpCAM** | Origene | TA506627 | Rabbit | 1:400 |
| **PROCR** | R＆D systems | MAB22451 | mouse | 1:150 |
| **Twist** | Abcam | Ab175430 | Mouse | 1:100 |
| **Slug** | CST | C19G7 | Rabbit | 1:400 |
